# Supplementary figures and images for: The co-receptor Tetraspanin12 directly captures Norrin to promote ligand-specific β-catenin signaling
Source: eLife. 2025 Jan 2;13:RP96743. doi: 10.7554/eLife.96743 (PMC11695057; doi:10.7554/eLife.96743)

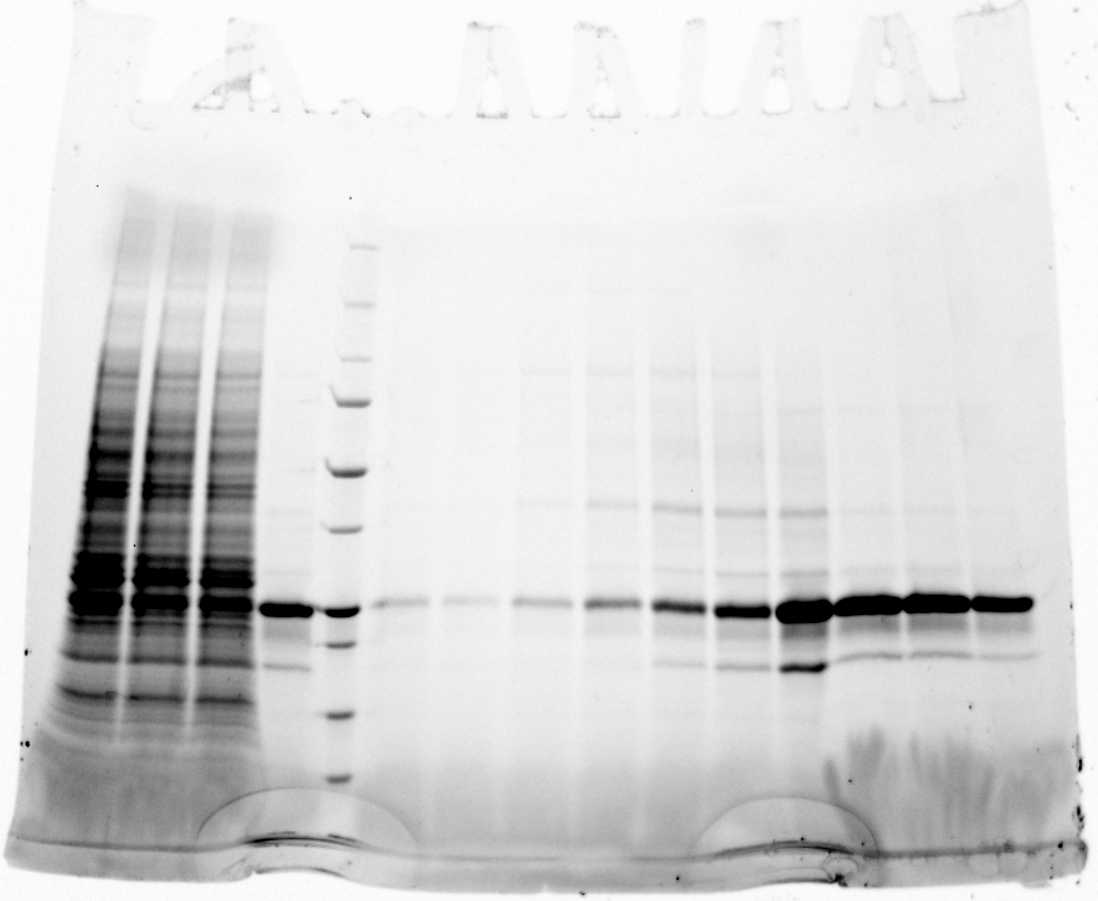

Supplement: Figure 1—figure supplement 1—source data 1. [file elife-96743-fig1-figsupp1-data1.zip › figure 1 figure supplement 1 original files/figure 1ΓÇöfigure supplement 1C.tif]

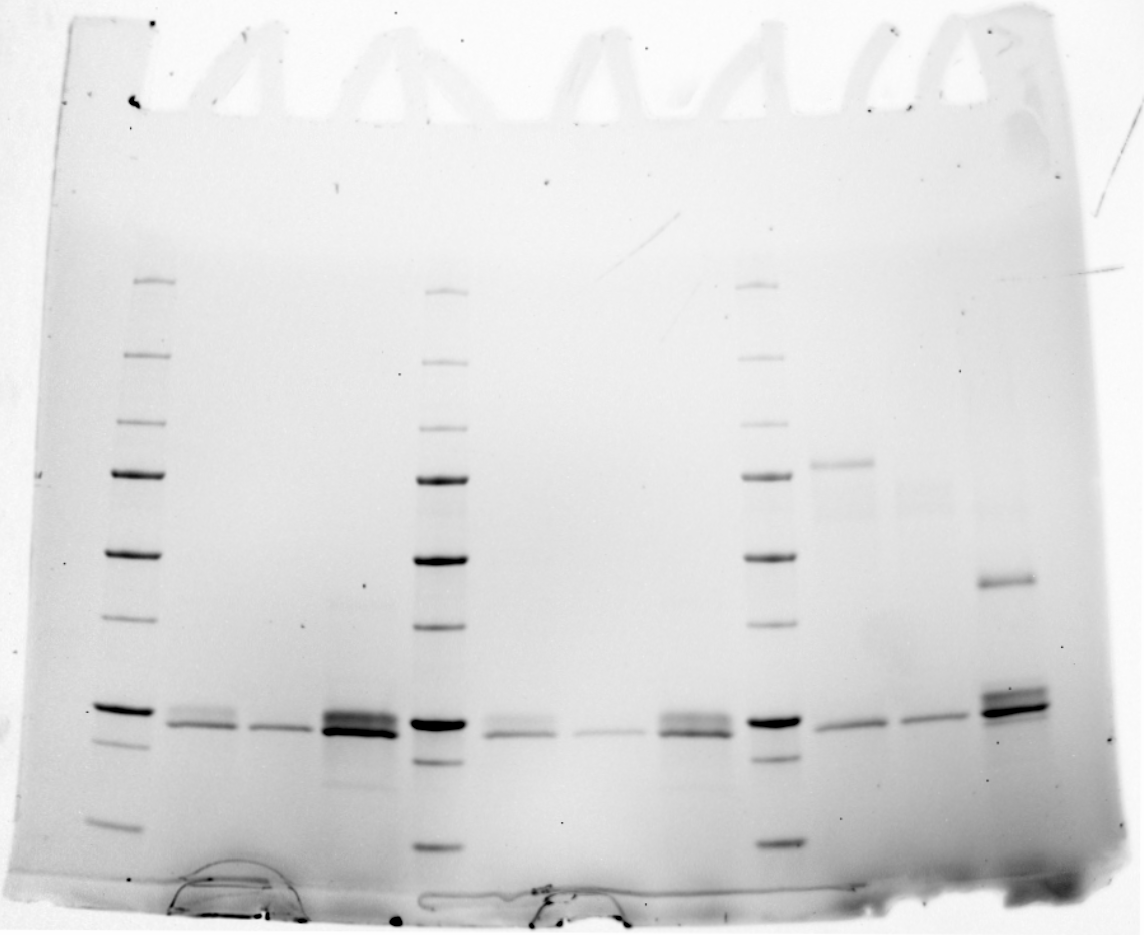

Supplement: Figure 1—figure supplement 1—source data 1. [file elife-96743-fig1-figsupp1-data1.zip › figure 1 figure supplement 1 original files/figure 1ΓÇöfigure supplement 1E.tif]

**figure 1—figure supplement 1E**

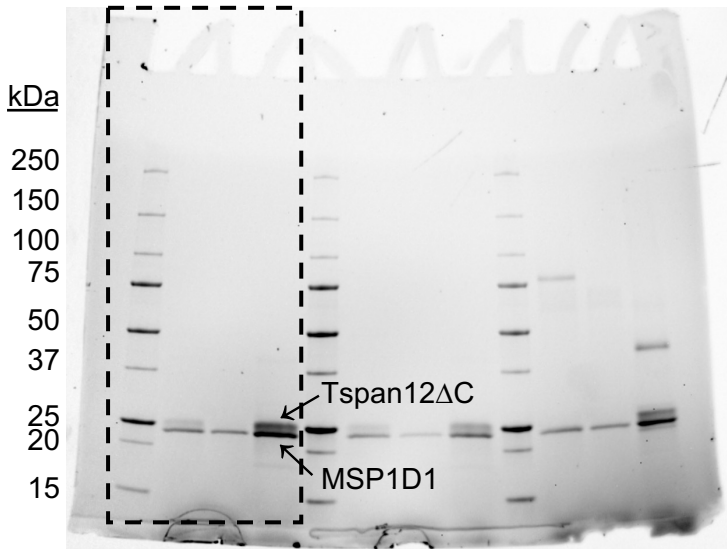

Supplement: Figure 1—figure supplement 1—source data 2. [file elife-96743-fig1-figsupp1-data2.zip › figure 1 figure supplement 1 original files labeled/figure 1ΓÇöfigure supplement 1E.pdf]

**figure 1—figure supplement 1C**

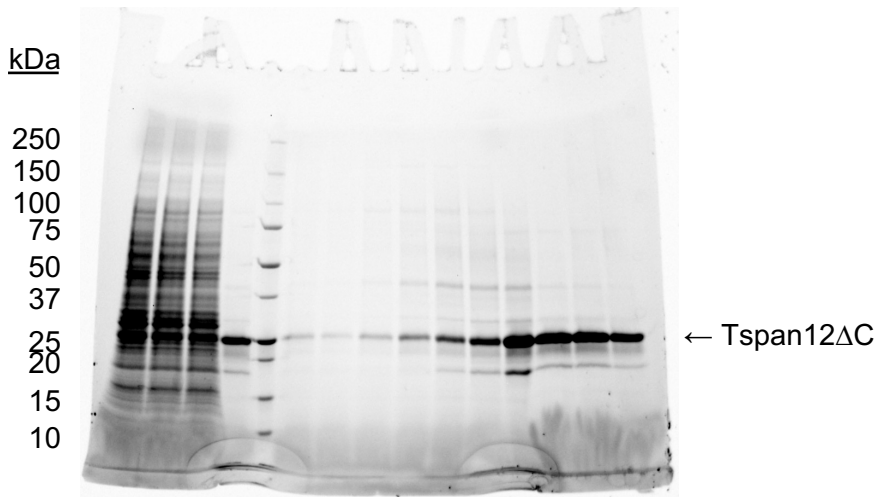

Supplement: Figure 1—figure supplement 1—source data 2. [file elife-96743-fig1-figsupp1-data2.zip › figure 1 figure supplement 1 original files labeled/figure 1ΓÇöfigure supplement 1C.pdf]

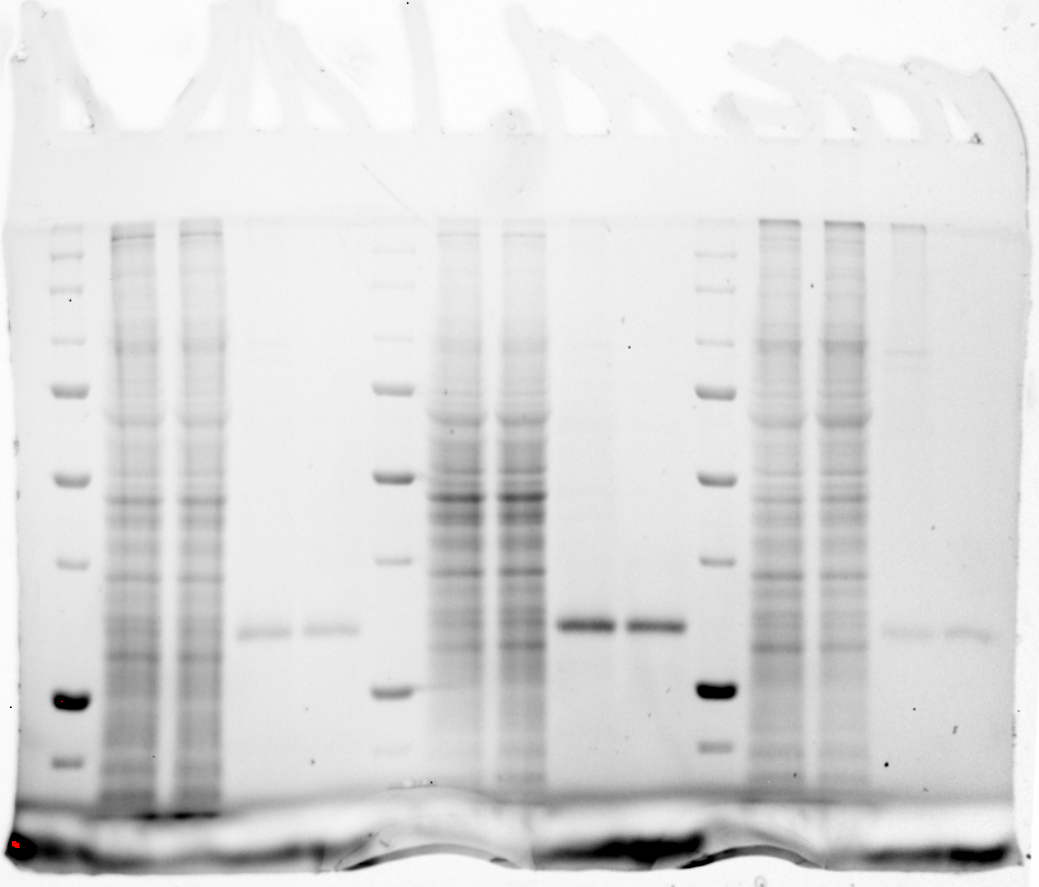

Supplement: Figure 2—figure supplement 2—source data 1. [file elife-96743-fig2-figsupp2-data1.zip › figure 2 figure supplement 2 original files/figure 2ΓÇöfigure supplement 2B-right.tif]

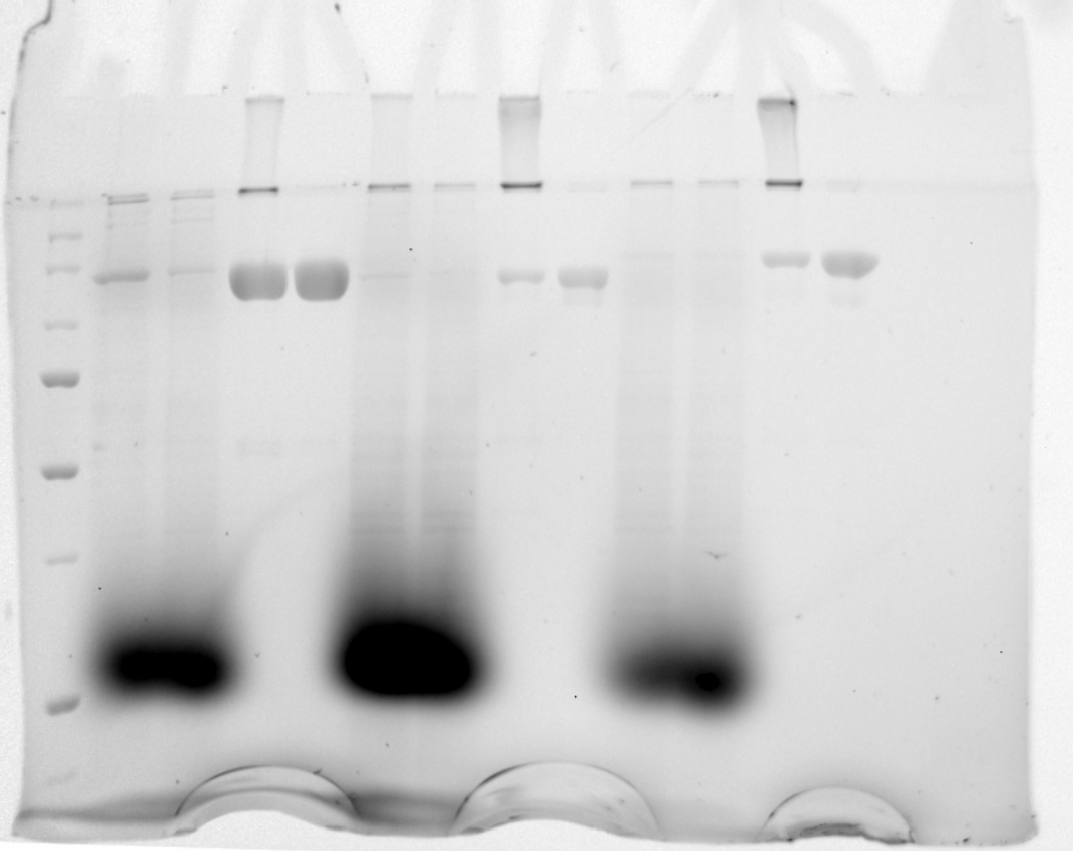

Supplement: Figure 2—figure supplement 2—source data 1. [file elife-96743-fig2-figsupp2-data1.zip › figure 2 figure supplement 2 original files/figure 2ΓÇöfigure supplement 2A-left.tif]

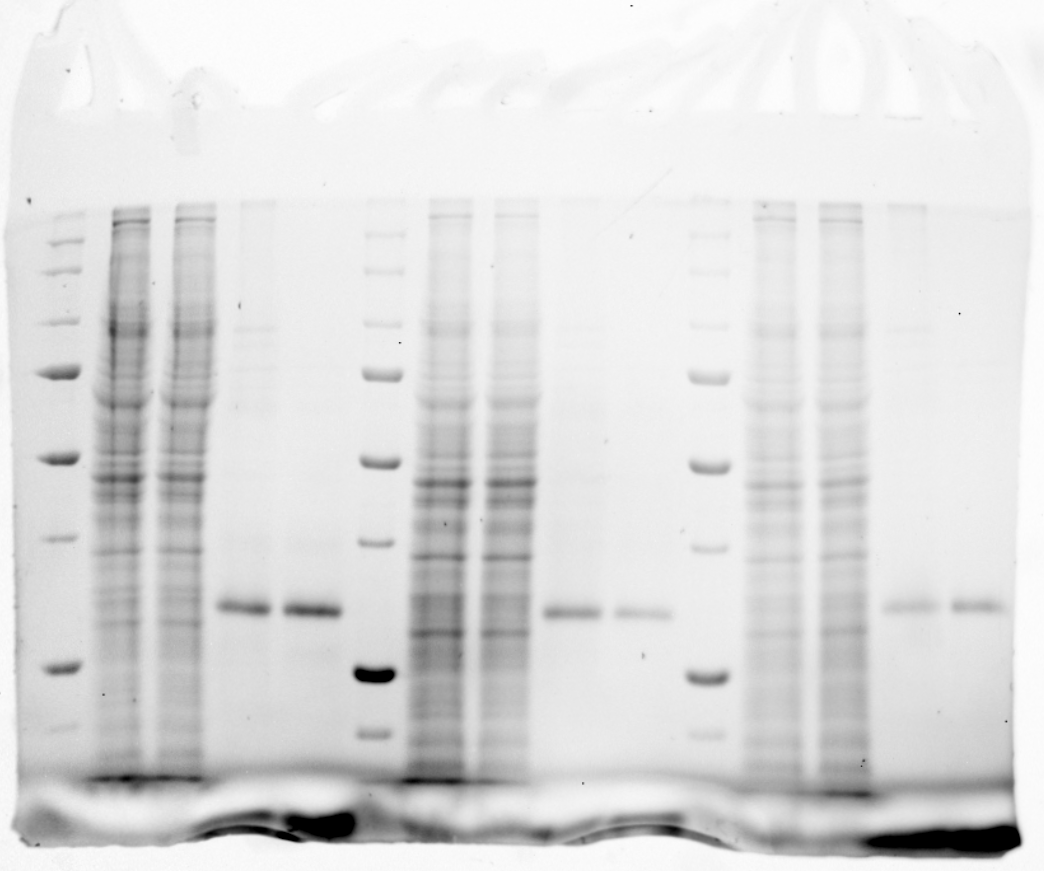

Supplement: Figure 2—figure supplement 2—source data 1. [file elife-96743-fig2-figsupp2-data1.zip › figure 2 figure supplement 2 original files/figure 2ΓÇöfigure supplement 2B-left.tif]

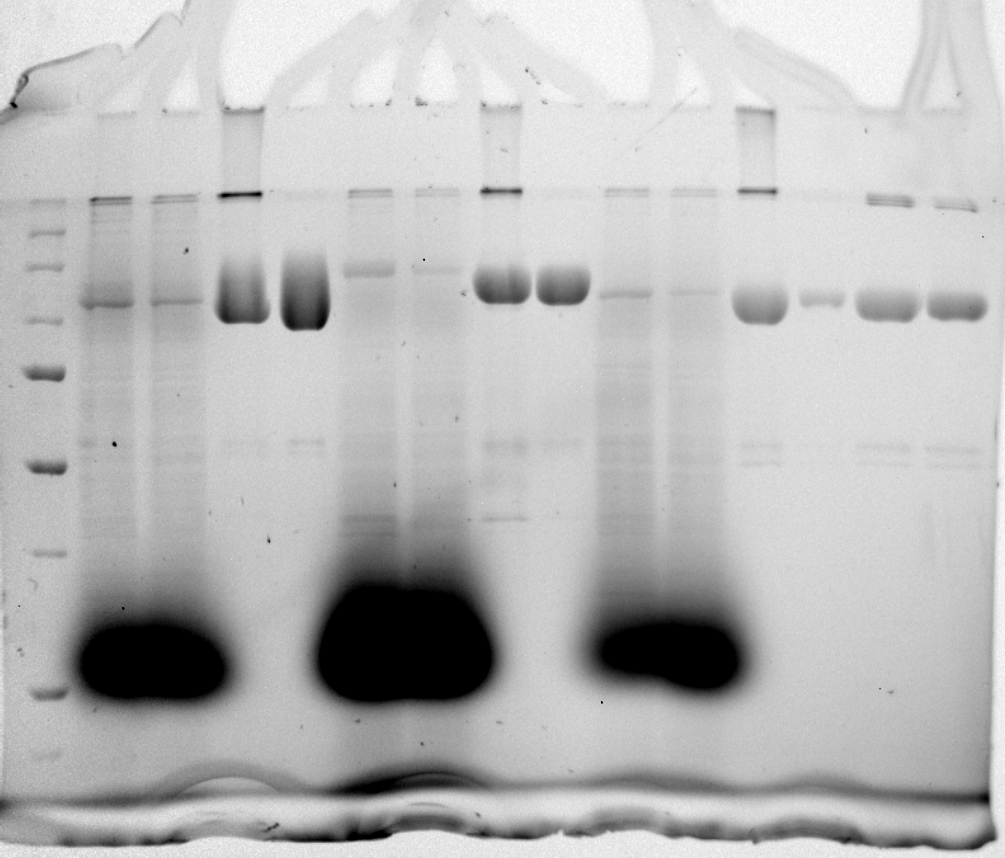

Supplement: Figure 2—figure supplement 2—source data 1. [file elife-96743-fig2-figsupp2-data1.zip › figure 2 figure supplement 2 original files/figure 2ΓÇöfigure supplement 2A-right.tif]

**figure 2—figure supplement 2A-left**

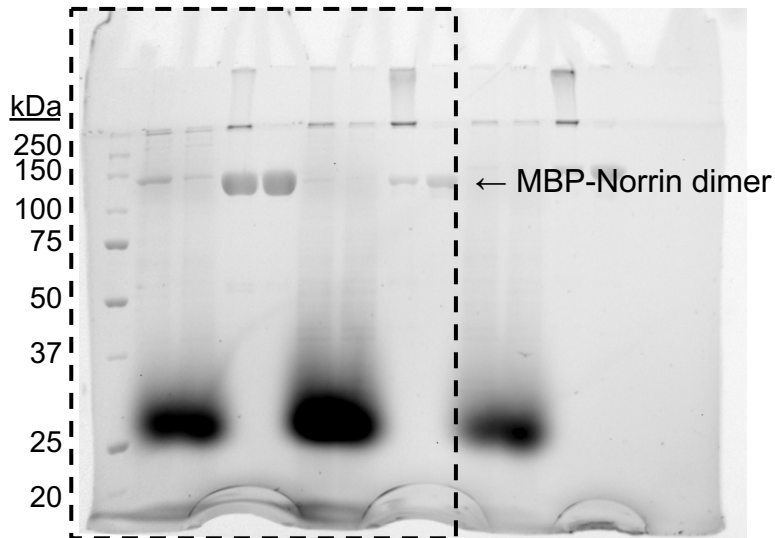

Supplement: Figure 2—figure supplement 2—source data 2. [file elife-96743-fig2-figsupp2-data2.zip › figure 2 figure supplement 2 original files labeled/figure 2ΓÇöfigure supplement 2A-left.pdf]

**figure 2—figure supplement 2B-right**

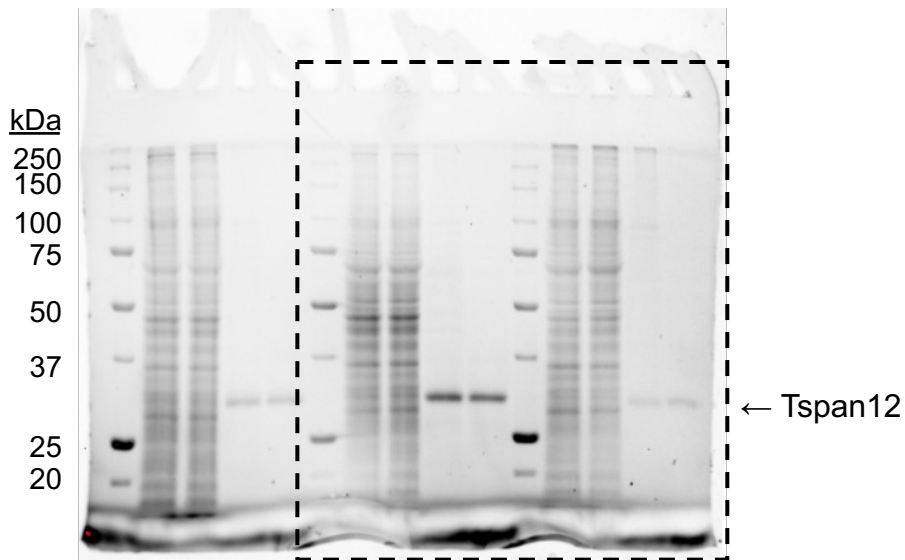

Supplement: Figure 2—figure supplement 2—source data 2. [file elife-96743-fig2-figsupp2-data2.zip › figure 2 figure supplement 2 original files labeled/figure 2ΓÇöfigure supplement 2B-right.pdf]

**figure 2—figure supplement 2A-right**

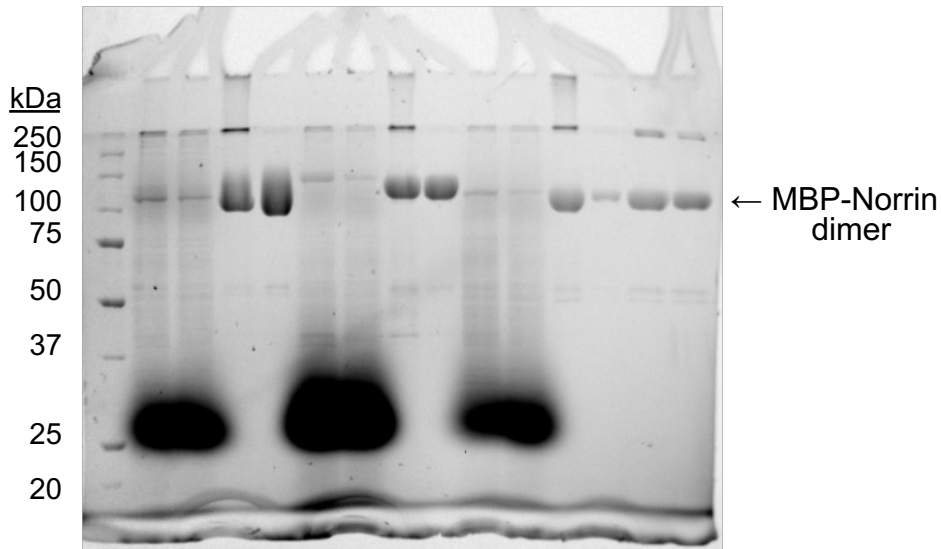

Supplement: Figure 2—figure supplement 2—source data 2. [file elife-96743-fig2-figsupp2-data2.zip › figure 2 figure supplement 2 original files labeled/figure 2ΓÇöfigure supplement 2A-right.pdf]

**figure 2—figure supplement 2B-left**

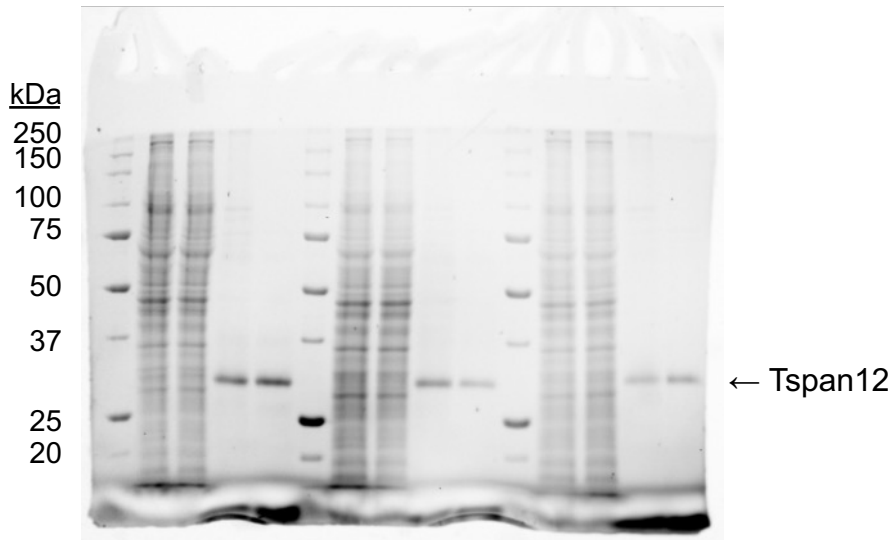

Supplement: Figure 2—figure supplement 2—source data 2. [file elife-96743-fig2-figsupp2-data2.zip › figure 2 figure supplement 2 original files labeled/figure 2ΓÇöfigure supplement 2B-left.pdf]

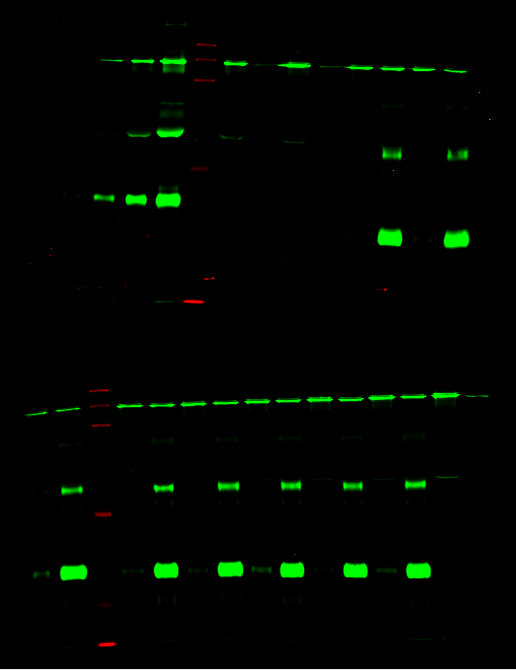

Supplement: Figure 3—figure supplement 1—source data 1. [file elife-96743-fig3-figsupp1-data1.zip › figure 3 figure supplement 1 original files/figure 3ΓÇöfigure supplement 1B.tif]

**figure 3—figure supplement 1B**

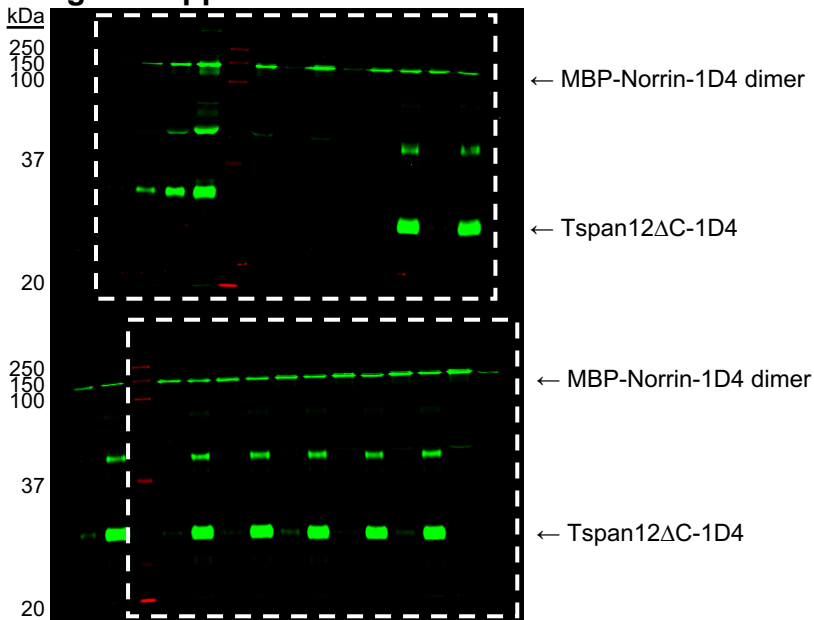

Supplement: Figure 3—figure supplement 1—source data 2. [file elife-96743-fig3-figsupp1-data2.zip › figure 3 figure supplement 1 original files labeled/figure 3ΓÇöfigure supplement 1B.pdf]

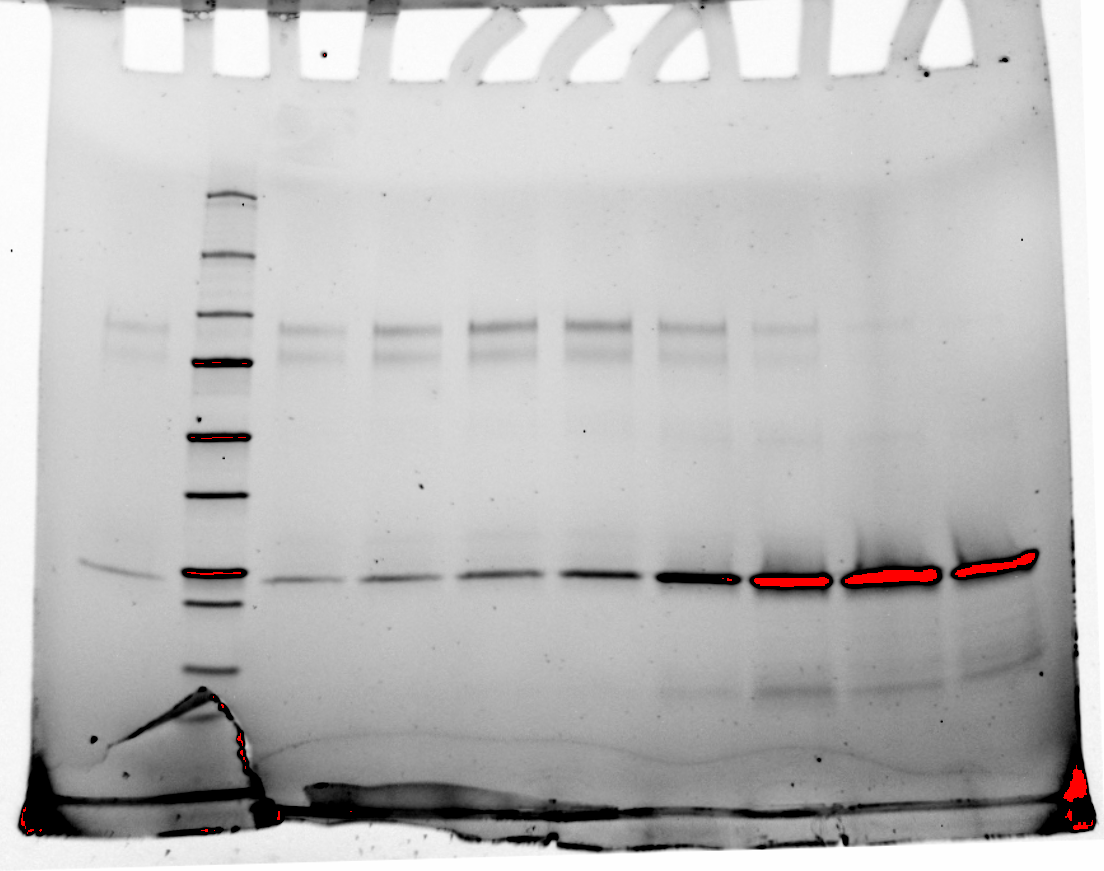

Supplement: Figure 4—figure supplement 1—source data 1. [file elife-96743-fig4-figsupp1-data1.zip › figure 4 figure supplement 1 original files/figure 4ΓÇöfigure supplement 1D-upper.tif]

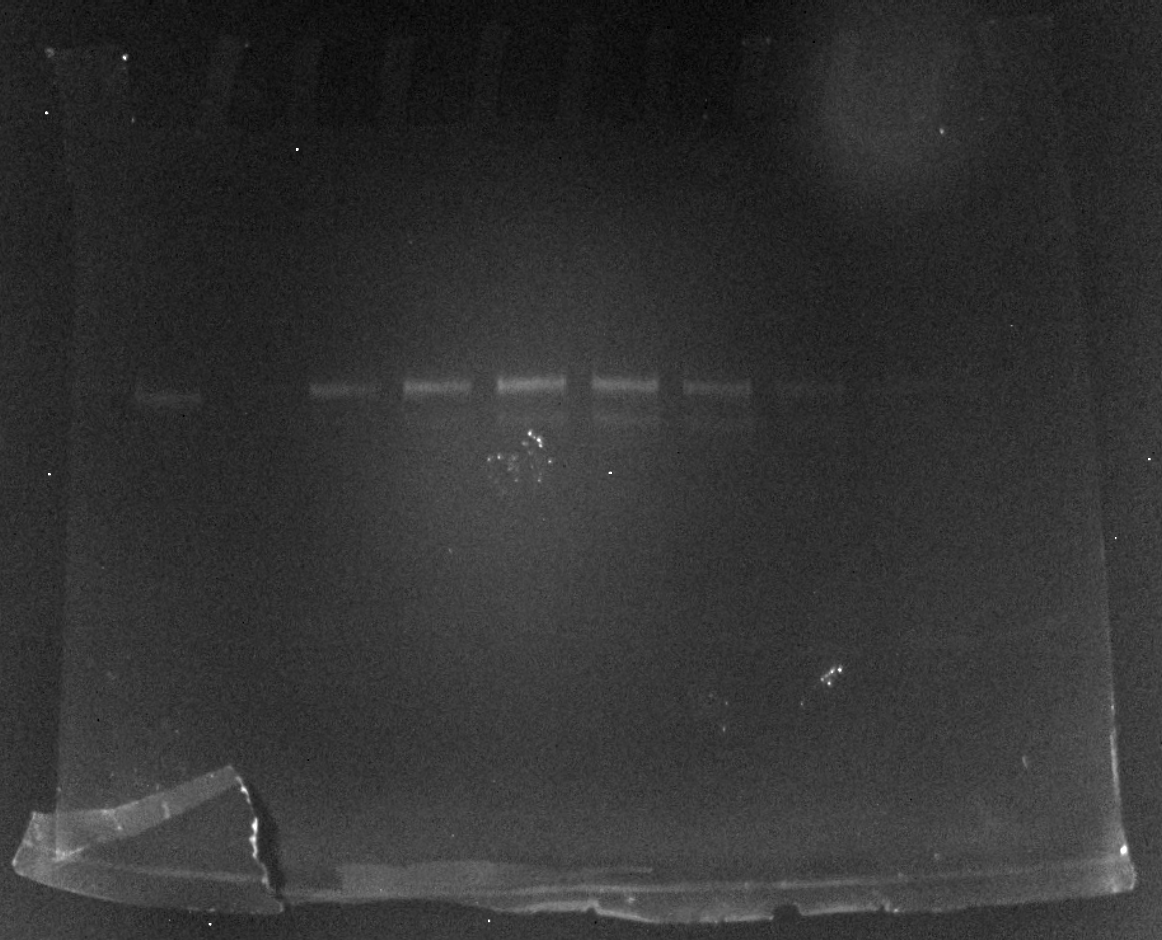

Supplement: Figure 4—figure supplement 1—source data 1. [file elife-96743-fig4-figsupp1-data1.zip › figure 4 figure supplement 1 original files/figure 4ΓÇöfigure supplement 1D-lower.tif]

figure 4—figure supplement 1D-lower

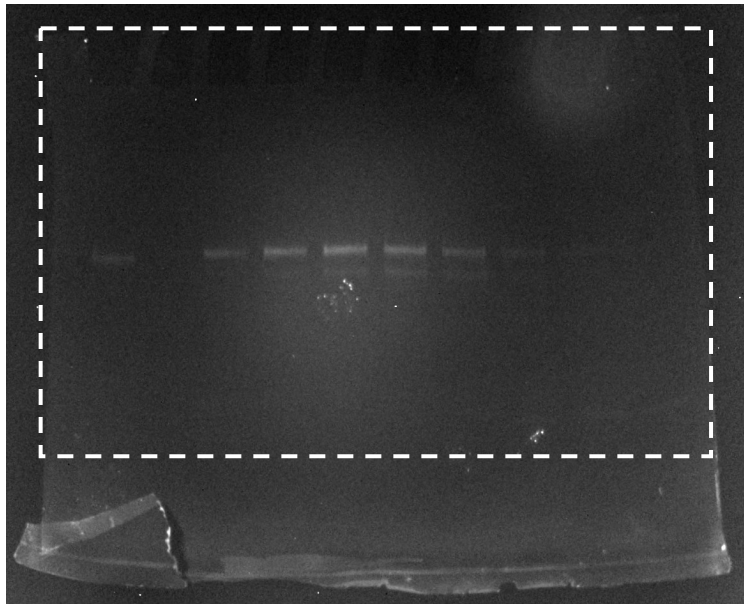

Tspan12/  
← Fzd4-SGFP

Supplement: Figure 4—figure supplement 1—source data 2. [file elife-96743-fig4-figsupp1-data2.zip › figure 4 figure supplement 1 original files labeled/figure 4ΓÇöfigure supplement 1D-lower.pdf]

figure 4—figure supplement 1D-upper

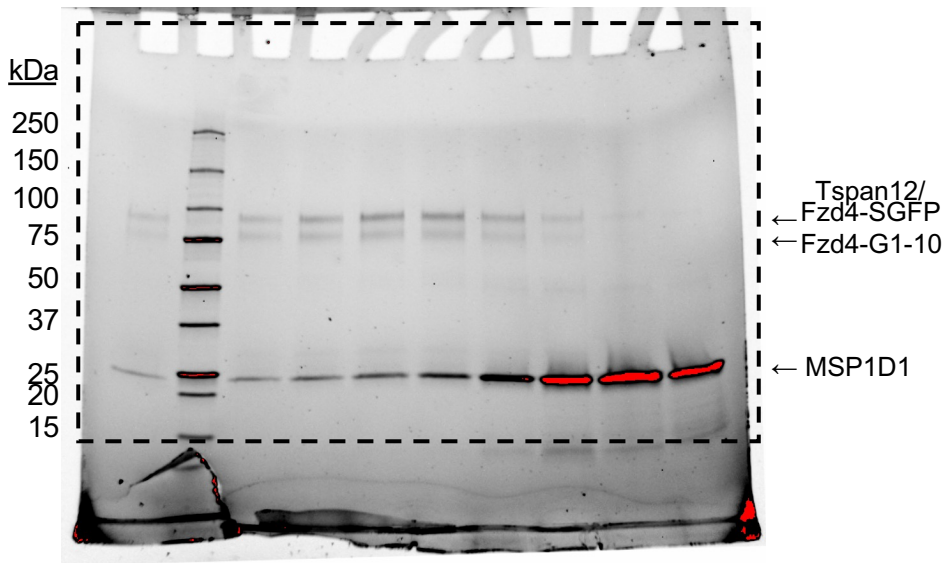

Supplement: Figure 4—figure supplement 1—source data 2. [file elife-96743-fig4-figsupp1-data2.zip › figure 4 figure supplement 1 original files labeled/figure 4ΓÇöfigure supplement 1D-upper.pdf]

**figure 4—figure supplement 1E**

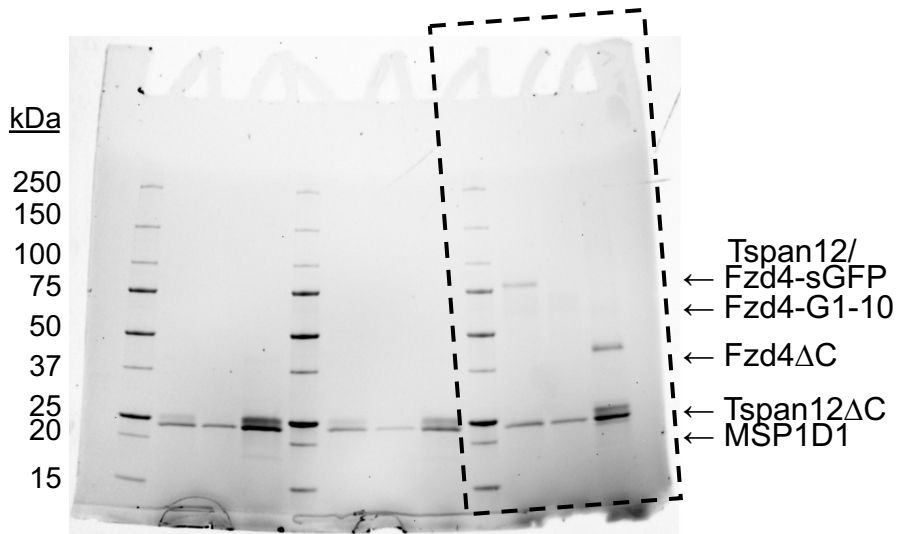

Supplement: Figure 4—figure supplement 1—source data 2. [file elife-96743-fig4-figsupp1-data2.zip › figure 4 figure supplement 1 original files labeled/figure 4ΓÇöfigure supplement 1E.pdf]

**figure 4—figure supplement 2C-middle**

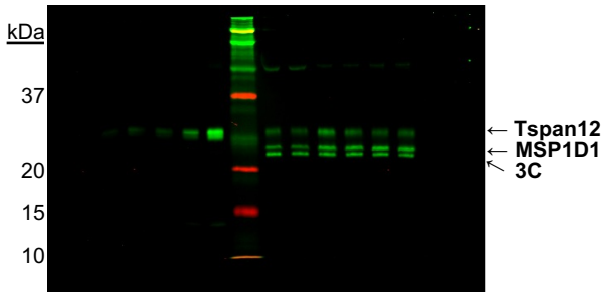

Supplement: Figure 4—figure supplement 2—source data 2. [file elife-96743-fig4-figsupp2-data2.zip › figure 4 figure supplement 2 original files labeled/figure 4ΓÇöfigure supplement 2C-middle.pdf]

**figure 4—figure supplement 2C-lower**

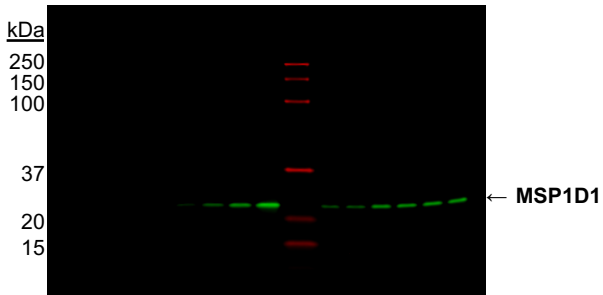

Supplement: Figure 4—figure supplement 2—source data 2. [file elife-96743-fig4-figsupp2-data2.zip › figure 4 figure supplement 2 original files labeled/figure 4ΓÇöfigure supplement 2C-lower.pdf]

**figure 4—figure supplement 2B-upper**

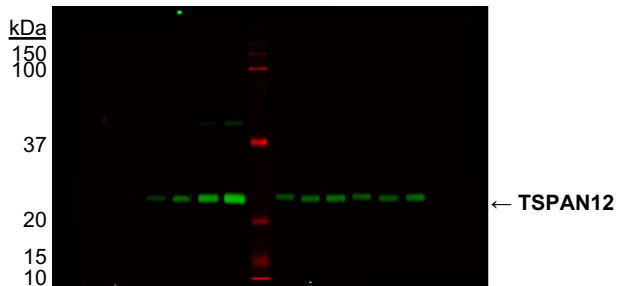

Supplement: Figure 4—figure supplement 2—source data 2. [file elife-96743-fig4-figsupp2-data2.zip › figure 4 figure supplement 2 original files labeled/figure 4ΓÇöfigure supplement 2B-upper.pdf]

**figure 4—figure supplement 2B-lower**

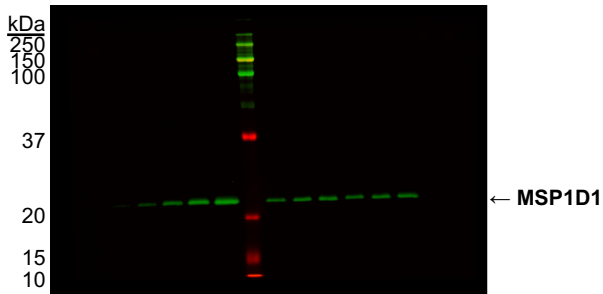

Supplement: Figure 4—figure supplement 2—source data 2. [file elife-96743-fig4-figsupp2-data2.zip › figure 4 figure supplement 2 original files labeled/figure 4ΓÇöfigure supplement 2B-lower.pdf]

**figure 4—figure supplement 2C-upper**

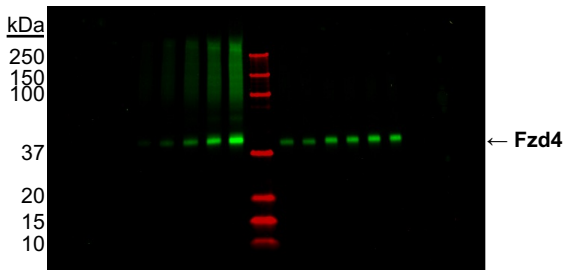

Supplement: Figure 4—figure supplement 2—source data 2. [file elife-96743-fig4-figsupp2-data2.zip › figure 4 figure supplement 2 original files labeled/figure 4ΓÇöfigure supplement 2C-upper.pdf]

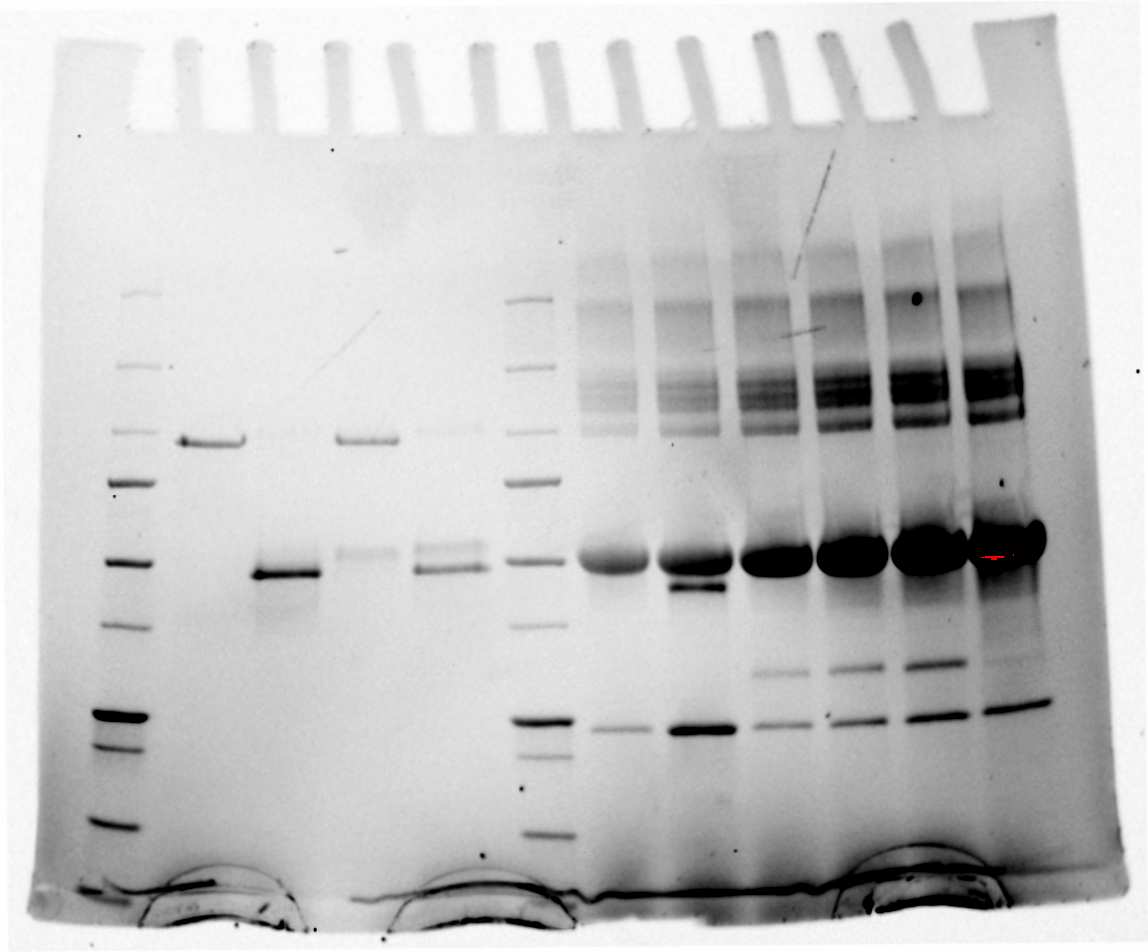

Supplement: Figure 4—figure supplement 4—source data 1. [file elife-96743-fig4-figsupp4-data1.zip › figure 4 figure supplement 4 original files/figure 4ΓÇöfigure supplement 4B.tif]

**figure 4—figure supplement 4B**

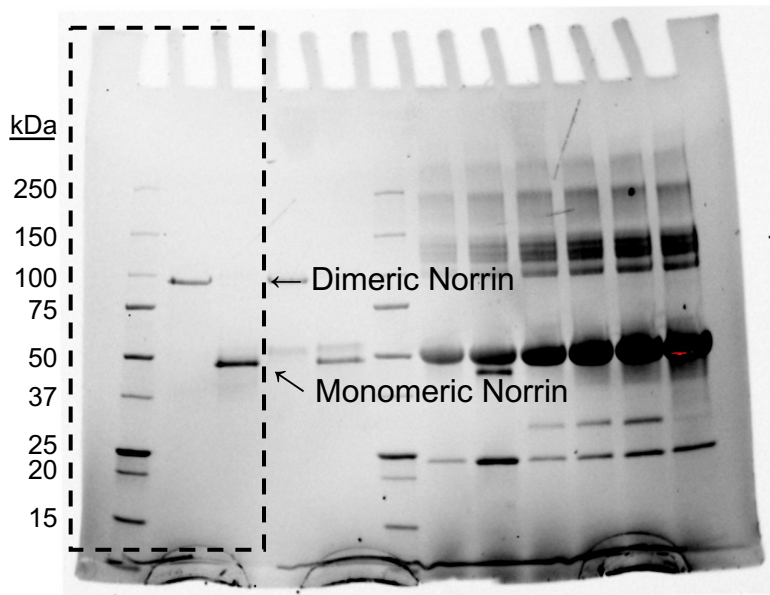

Supplement: Figure 4—figure supplement 4—source data 2. [file elife-96743-fig4-figsupp4-data2.zip › figure 4 figure supplement 4 original files labeled/figure 4ΓÇöfigure supplement 4B.pdf]

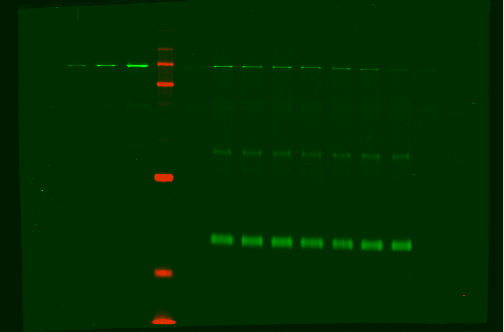

Supplement: Figure 5—figure supplement 1—source data 1. [file elife-96743-fig5-figsupp1-data1.zip › figure 5 figure supplement 1 original files/figure 5ΓÇöfigure supplement 1A.tif]

**figure 5—figure supplement 1A**

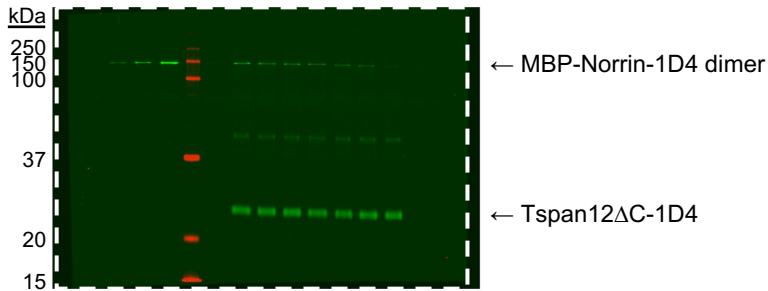

Supplement: Figure 5—figure supplement 1—source data 2. [file elife-96743-fig5-figsupp1-data2.zip › figure 5 figure supplement 1 original files labeled/figure 5ΓÇöfigure supplement 1A.pdf]
