# Supplementary material for: The co-receptor Tetraspanin12 directly captures Norrin to promote ligand-specific β-catenin signaling
Source: eLife. 2025 Jan 2;13:RP96743. doi: 10.7554/eLife.96743 (PMC11695057; doi:10.7554/eLife.96743)
Supplement: Supplementary file 1. [file elife-96743-supp1.docx]

**Supplementary file 1.** Table of kinetic constants and steady state affinities quantified by biolayer interferometry.

| **Bait (biosensor)** | **Analyte  (in solution)** | **Steady- state  K_D_ (nM)** | **Kinetic K_D_ (nM)** | **K_on_ (nM ^-1^ s^-1^) x 10^-4^** | **K_off_ (s^-1^)  x 10^-4^** | **Relating to figure** | **Notes** |
| --- | --- | --- | --- | --- | --- | --- | --- |
| Tspan12∆C | Norrin | 10.4 ± 1.2 | 7.4 ± 1.4 | 1.9 ± 0.3 | 14 ± 2 | 1A-D, 4A | *h* = 0.98 ± 0.1 |
| MBP-Norrin | Tspan12 LEL | 17.9 ± 3.0 | 16 ± 3 | 0.70 ± 0.08 | 11 ± 2 | 1E |  |
| Tspan12-LEL11 | Norrin |  | n.b. at 100 nM |  |  | 1F |  |
| MBP-Norrin | Tspan12∆C  in nanodiscs | 18.0 ± 4.0 | 34 ± 11 | 1.0 ± 0.3 | 34 ± 3 | 1.s2A |  |
| MBP-Norrin | Tspan12∆C  in GDN |  | n.d.  ( > 100 nM) |  |  | 1.s2B |  |
| Fzd4 | Tspan12 LEL (MBP-tagged) |  | n.b.  at 18 µM |  |  | 1.s2C |  |
| Tspan12∆C | Fzd4 CRDL |  | n.b.  at 32 µM |  |  | 1.s2D |  |
| MBP-Norrin | Fzd4 CRDL | 122 ± 38 | 340 ± 42 | 0.43 ± 0.05 | 150 ± 10 | 3.s1C |  |
| Tspan12∆C | WT Norrin |  | 2.9 ± 0.7 | 1.9 ± 0.5 | 4.8 ± 0.5 |  | Measured at 32 nM Norrin |
| WT Tspan12 | WT Norrin |  | 2.5 ± 0.2 | 1.1 ± 0.1 | 2.7 ± 0.2 | 2, 2.s3 | n = 6;  Measured at 32 nM Norrin |
| WT Tspan12 | Norrin R107E/R115E |  | 210 ± 24 | 1.9 ± 0.5 | 520 ± 30 | 2E, 2.s3 | Measured at 32 nM Norrin |
| WT Tspan12 | Norrin T117Y/T119Y |  | 30 ± 7 | 0.32 ± 0.02 | 9.6 ± 1.9 | 2E, 2.s3 | Measured at 32 nM Norrin |
| WT Tspan12 | Norrin K102E/R121E |  | 240 ± 28 | 1.7 ± 0.3 | 380 ± 40 | 2E, 2.s3 | Measured at 32 nM Norrin |
| WT Tspan12 | Norrin S82D |  | 5.4 ± 0.4 | 1.5 ± 0.2 | 8.4 ± 1.5 | 2E, 2.s3 | Measured at 32 nM Norrin |
| Tspan12 E173K/D175K | WT Norrin |  | 8.5 ± 1.2 | 0.75 ± 0.07 | 6.3 ± 0.3 | 2F, 2.s3 | Measured at 32 nM Norrin |
| Tspan12 L198Y | WT Norrin |  | 1.9 ± 0.16 | 0.95 ± 0.05 | 1.7 ± 0.1 | 2F, 2.s3 | Measured at 32 nM Norrin |
| Tspan12 E196K/S199K | WT Norrin |  | 14 ± 1.1 | 0.70 ± 0.09 | 9.5 ± 0.4 | 2F, 2.s3 | Measured at 32 nM Norrin |
| Tspan12 E170K | WT Norrin |  | 16 ± 1.3 | 0.66 ± 0.03 | 10 ± 0.6 | 2F, 2.s3 | Measured at 32 nM Norrin |
| Tspan12 E173K/D175K | Norrin R107E/R115E |  | n.b.  at 32 nM |  |  | 2.s4 | Measured at 32 nM Norrin |
| Tspan12 L198Y | Norrin T117Y/T119Y |  | 23 ± 4.6 | 0.34 ± 0.09 | 8.7 ± 4.0 | 2.s4 | Measured at 32 nM Norrin |
| Tspan12 E196K/S199K | Norrin K102E/R121E |  | 1.3 ± 0.06 | 6.7 ± 0.3 | 8.8 ± 0.2 | 2.s4 | Measured at 32 nM Norrin |
| Tspan12 E170K | Norrin S82D |  | 5.2 ± 0.7 | 1.5 ± 0.03 | 7.7 ± 1.2 | 2.s4 | Measured at 32 nM Norrin |
| Fzd4 | Norrin dimer | 3.29 ± 0.17 | n.d. | 3.1 ± 0.4 | 10 ± 1 *^a^* | 4A, 4.s3A | *h* = 2.8 ± 0.6 |
| Tspan12/Fzd4 dimer | Norrin dimer | 2.18 ± 0.10 | n.d. | 4.3 ± 0.6 | 9.5 ± 1.0 *^a^* | 4A, 4.s3B | *h* = 1.7 ± 0.1 |
| Tspan12∆C | Norrin monomer | 820 ± 100 | 80 ± 35 | 0.52 ± 0.20 | 41 ± 9 | 4B | *h* = 1.0 ± 0.1 |
| Fzd4 | Norrin monomer | 13.2 ± 1.2 | n.d. | 1.9 ± 0.3 | 26 ± 4 *^a^* | 4B, 4.s3D | *h* = 2.0 ± 0.3 |
| Tspan12/Fzd4 dimer | Norrin monomer | 11.5 ± 1.5 | n.d. | 1.9 ± 0.3 | 22 ± 4 *^a^* | 4B, 4.s3E | *h* = 1.0 ± 0.1 |
| Fzd4*^b^* | Dvl2 DEP | 183 ± 24 | 220 ± 28 | 0.62 ± 0.07 | 140 ± 7 | 5B-C |  |
| Tspan12/Fzd4 dimer *^b^* | Dvl2 DEP | 279 ± 46 | 280 ± 49 | 0.45 ± 0.07 | 130 ± 6 | 5C |  |
| Fzd4 *^b^* + Norrin | Dvl2 DEP | 161 ± 21 | 160 ± 13 | 0.82 ± 0.06 | 130 ± 4 | 5D |  |
| Tspan12/Fzd4 dimer *^b^* + Norrin | Dvl2 DEP | 274 ± 39 | 280 ± 110 | 0.43 ± 0.17 | 120 ± 6 | 5D |  |

Note: Values represent mean ± S.E.M. for n=3 independent experiments unless otherwise noted.
Steady state K_D_ was determined for experiments in which ≥ 3 different concentrations of analyte were measured.

Bait receptors (Tspan12, Fzd4, and Tspan12/Fzd4) were embedded in MSP1D1 nanodiscs (48:32:20 POPC:POPG:Cholesterol) unless otherwise noted.
Abbreviations: n.b. = no binding. n.d. = not determined, i.e. fit was of low confidence.; *h* = hill coefficient.
*^a^* K_off_ determined by steady state K_D_*K_on_, not by direct fitting of dissociation traces due to fits of low confidence.
*^b^* For Dvl2 DEP binding, the bait receptor was inserted into MSP1E3D1 nanodiscs (70:5:20 POPC:PI(4,5)P_2_:Cholesterol)
